# Between innovation and risk: artificial intelligence and data protection in digital Mexico

**DOI:** 10.3389/frai.2026.1716108

**Published:** 2026-05-13

**Authors:** Emilio J. Medrano-Sánchez, Elizabeth Ruiz-Ramírez, Mariela L. Ayllon

**Affiliations:** 1 Universidad San Ignacio de Loyola, Lima, Peru; 2 Universidad Nacional Autónoma de México, Mexico City, Mexico; 3 Universidad Tecnológica del Perú, Ica, Peru

**Keywords:** artificial intelligence, citizen trust, digital public policy, personal data protection, social perception

## Abstract

At a global level, the rapid adoption of artificial intelligence (AI) brings significant risks to data privacy, prompting the development of legal frameworks. In Latin America, including Mexico, where such frameworks remain emergent, the issue gains particular relevance. This study analyzed how perceptions of AI are associated with perceptions of personal data protection among university-educated professionals residing in Mexico City in 2025, based on privacy theory, the Technology Acceptance Model (TAM), and their extensions. A quantitative, non-experimental, correlational, and cross-sectional approach was employed. Data were collected from 101 university-educated professional participants residing in Mexico City using an expert-validated, 24-item Likert-type questionnaire. Data analysis was conducted in SPSS using non-parametric correlation tests (Spearman and Kendall). Results indicated that a more favorable perception of AI was associated with more favorable perceptions of personal data protection and its dimensions. The correlations demonstrated a moderate and significant positive association (*p* < 0.001) between perceptions of AI and overall perceptions of data protection. Furthermore, significant positive correlations were found across each evaluated dimension, thus confirming the four specific hypotheses. From a theoretical perspective, the findings suggest that contextual factors modulate the AI-privacy relationship, contributing a Latin American perspective to the literature. On a practical and social level, recommendations aligned with SDG 16 include strengthening institutional frameworks (regulation, transparency, digital education), fostering public-private collaboration, and promoting digital oversight and literacy to achieve informed trust.

## 1 Introduction

The adoption of artificial intelligence (AI) is growing at an unprecedented rate. Currently, 35% of organizations employ AI, another 42% are evaluating its use, and by 2024 more than half plan to integrate it into their processes; in absolute terms, at least 250 million companies are already using or investigating AI technologies (Richard et al., 2025). While this massive deployment enhances operational efficiency, it also increases risks for data privacy and security (see Table 1). AI tools are commonly employed to strengthen cyber defense; however, their inherent sophistication can also be exploited by attackers, leading to significant data breaches (Khder et al., 2023; Morovat and Panda, 2020). Among the most cited dangers are leaks of sensitive data (He et al., 2025), malicious code injection (Pelekis et al., 2025), and intrusive user profiling (Kamaruddin et al., 2021; Mach, 2025).

**Table 1:** Overview of the cybersecurity landscape and AI adoption.

Author	Aspect	Details
Durgadevi et al. (2024)	Average cost per breach	USD 3.86 million
Durgadevi et al. (2024)	Average recovery time	196 days
Jadhav et al. (2024)	Cost increase (2020–2022)	13%
Botha et al. (2017)	Total breaches (2005–2021)	901 million
Richard et al. (2025)	Use of AI in cybersecurity	35% adoption; 77% trust its potential
Attaallah et al. (2022); Samonte et al. (2024); Uwizeyemungu and Poba-Nzaou, 2020	Healthcare sector breaches	Increasing at an alarming rate
Samonte et al. (2024)	Key prevention strategies	AI-based IDS, IAM protocols, encryption
Singh (2025); Pelekis et al. (2025)	Trends	From simple attacks to sophisticated AI-driven cyber threats

Against this backdrop, various regulatory frameworks seek to balance innovation with safeguards. Europe's General Data Protection Regulation (GDPR) mandates transparency and limits on automated data processing (Al-Tkhayneh et al., 2023), while the California Consumer Privacy Act (CCPA) establishes similar rights within the United States (Burton et al., 2024). Simultaneously, the forthcoming European Union AI Act aims to impose specific obligations on developers and users of intelligent systems (Dunphy-Moriel and Berton, 2025; Hayes, 2022). The same AI and machine learning technologies that pose new challenges also provide unprecedented capabilities for anomaly detection and breach prevention by analyzing large datasets (Candan and Kalay, 2024). Recent literature emphasizes complementing these regulations with ethical principles and proactive measures that prioritize social welfare (Kashiramka et al., 2024; Mazzi et al., 2023; Wirtz et al., 2022). Furthermore, comparative studies of BRICS nations reveal disparities, underscoring the importance of robust data protection legislation in emerging economies (Sharma and Sharma, 2024).

The global agenda described acquires specific nuances in Latin America, where the expansion of AI intersects with heterogeneous and often incipient data protection frameworks (see Table 2). In Mexico, privacy legislation is relatively recent and still undergoing consolidation; significant gaps remain compared to the European GDPR, especially regarding uniform application of principles such as minimization and portability (Demetzou et al., 2023; Maqueo, 2016). Several Latin American countries have adopted regulations inspired by the GDPR, but the lack of harmonization among states, coupled with unique initiatives like the recognition of neuro-rights in Chile, reveals a regulatory mosaic whose effectiveness depends significantly on interstate coordination (Contreras, 2024; Demetzou et al., 2023; Ramiro, 2022).

**Table 2:** Cybersecurity landscape and AI adoption in Mexico and Latin America: comparative overview.

Aspect	Mexico (examples)	Latin America (examples)	Citations
Regulatory gaps	Evolving regulations; gaps compared to GDPR	GDPR-inspired; neuro-rights; lack of harmonization	(Contreras, 2024; Demetzou et al., 2023; Maqueo, 2016; Ramiro, 2022)
Cybersecurity rankings	National strategy; challenges addressing AI threats	Leadership by Brazil; disparities in ILIA and WAIN indexes	(Acosta-Vargas et al., 2025; Bolgov, 2020; Kosevich, 2022)
Emblematic cases	Google Mexico and right to erasure; health data regulation	Cases illustrating AI's impact on public management	(Filgueiras, 2023; Juárez-Merino, 2025; Maqueo, 2016)
Authors on legal issues	Comparative analysis of national AI policies	Role of AI in public administration and related challenges	(Filgueiras, 2023; Juárez-Merino, 2025)

Examples illustrate the regulatory, strategic, and legal landscape of AI and cybersecurity in Mexico and Latin America.

In the Mexican context, personal data protection for private-sector entities has been formally regulated since the enactment of the Federal Law on the Protection of Personal Data Held by Private Parties (LFPDPPP) in 2010, which established core principles, obligations, and the ARCO rights framework (access, rectification, cancellation, and opposition) for data subjects (Gobierno de México, 2010). However, the accelerated digitalization of public and private services, together with the emergence of artificial intelligence–driven data processing, exposed structural limitations in the original regulatory design. In response to these challenges, a substantial regulatory update was enacted in 2025, introducing strengthened obligations, oversight mechanisms, and provisions explicitly addressing automated processing and advanced data analytics (Gobierno de México, 2025). This regulatory evolution provides a critical backdrop for examining how citizens perceive data protection in contemporary AI-intensive environments.

The cybersecurity landscape reveals similar disparities. Mexico has a National Cybersecurity Strategy aimed at protecting critical infrastructure and the digital economy (Kosevich, 2022); however, analysts highlight the need to reinforce it with specific protocols against AI-driven threats (Bolgov, 2020). In contrast, Brazil, Chile, and Uruguay hold the top regional positions in the Latin American AI Index and the Web Accessibility Index, reflecting differences in innovation and digital inclusion (Acosta-Vargas et al., 2025; Kosevich, 2022).

Emblematic cases illustrate how these regulatory tensions materialize. Mexico's Federal Institute for Access to Information and Data Protection (IFAI) compelled Google Mexico to erase a citizen's health data, relying on criteria established by the Court of Justice of the European Union in the Google Spain vs. AEPD case (Maqueo, 2016). Meanwhile, impending regulations regarding clinical data in Mexico foresee strict penalties for healthcare providers that violate emerging provisions.

From an academic perspective, comparative studies on national AI strategies in Argentina, Brazil, Chile, Colombia, Mexico, and Uruguay show that the type of political regime and governance model significantly influence the robustness of AI policies (Filgueiras, 2023). Research centered on Mexico City, Santiago, and Buenos Aires emphasizes AI's transformative potential to enhance transparency and civic participation yet cautions about the persistent digital divide and the risks of indiscriminate surveillance (Juárez-Merino, 2025).

The implementation of smart city initiatives in Mexico City proceeds amid obstacles that test the technical and regulatory capacities of the capital. Technologically, infrastructure deficiencies persist large-scale sensor networks and data processing centers capable of handling massive data streams generated by AI systems are still unavailable (Efthymiou and Egleton, 2023; Kaginalkar et al., 2022). This vulnerability is amplified because the urban ecosystem connects numerous devices and public services, increasing the attack surface and demanding security measures that ensure data privacy and integrity (Braun et al., 2018; Islam and Karimipour, 2023). Concurrently, a considerable part of the city's administration operates legacy platforms whose architecture complicates integrating modern safeguards, resulting in fragmented data protection across disparate technologies (Xu and Guo, 2025).

The regulatory sphere is also lagging behind. Establishing rules governing automated decision-making, preventing biases, and defining legal responsibilities remains challenging. Local legislation, although inspired by international frameworks, has yet to adopt specific provisions for predictive AI scenarios or intensive information sharing among agencies (Juárez-Merino, 2025; Lal et al., 2025; Zhou and Kankanhalli, 2021). Data governance relies on the Open Data platform but lacks robust guidelines ensuring traceability and oversight of subsequent citizen data use (Braun et al., 2018; Sharma et al., 2025). Furthermore, the social dimension poses additional challenges: the adoption of smart services depends heavily on public trust; however, privacy concerns curb residents' willingness to share personal information. Simultaneously, the digital divide restricts equitable access to innovation benefits, threatening to deepen pre-existing inequalities (Juárez-Merino, 2025; McCrory, 2024). The convergence of these factors underscores the importance of exploring how university-educated professionals residing in Mexico City perceive artificial intelligence and personal data protection, and the necessity of proposing guidelines harmonizing technological advancement with fundamental rights (see Table 3).

**Table 3:** Challenges of AI and data protection in Mexico City.

Category	Challenges
Technological	Infrastructure limitations, data privacy and security, integration with legacy systems
Regulatory	Developing comprehensive regulatory frameworks, data governance
Social	Citizen engagement and trust, digital divide

The specialized literature agrees that citizen trust is a key enabler for any successful deployment of artificial intelligence (AI). Conceptual reviews on trust in AI indicate that social acceptance depends on perceiving systems as predictable, transparent, and legitimate, and highlight the need for more empirical studies to measure trust in specific contexts (Benk et al., 2025). Surveys conducted among populations in various countries have revealed that individuals value technological reliability, but their assessments are strongly influenced by social interpretations of AI's impact on daily life (Gerlich, 2023). Such perceptions become even more critical when algorithms process large volumes of personal data: research on smart cities warns of algorithmic discrimination risks and calls for transparency, community participation, and continuous audits to maintain public legitimacy (Alashqar et al., 2025). Additionally, literature on misinformation emphasizes the urgency for responsible AI policies to protect privacy and informational pluralism (Needhipathi et al., 2025).

Empirical research in Latin America remains scarce and reveals significant asymmetries: technical capacities and academic production related to AI are concentrated in a few countries, leaving significant evidence gaps concerning how AI technologies are perceived and trusted in specific urban contexts (Chavez and Vizuete-Sandoval, 2025). Even in pioneering cases such as Mexico City, where AI already transforms public services and processes, available research has focused primarily on operational benefits and generic challenges such as the digital divide, without quantifying the relationship between citizen perception, trust, and data protection (Juárez-Merino, 2025). In summary, the literature identifies trust as the backbone of algorithmic governance, documents recurring ethical concerns, and emphasizes the need for inclusive regulatory frameworks. However, a noticeable empirical gap persists regarding the correlation between positive valuation of AI and privacy expectations in Mexico City. Therefore, this research specifically addresses this gap by providing data to measure the link between technological trust and personal data protection in the capital city's urban context.

From another international perspective, the study aligns with Sustainable Development Goal (SDG) 16 by offering inputs that promote transparency, institutional accountability, and effective protection of fundamental freedoms, presenting arguments for local governments to strengthen open information access mechanisms and ensure citizen participation in defining ethical AI frameworks.

**Figure 1:**
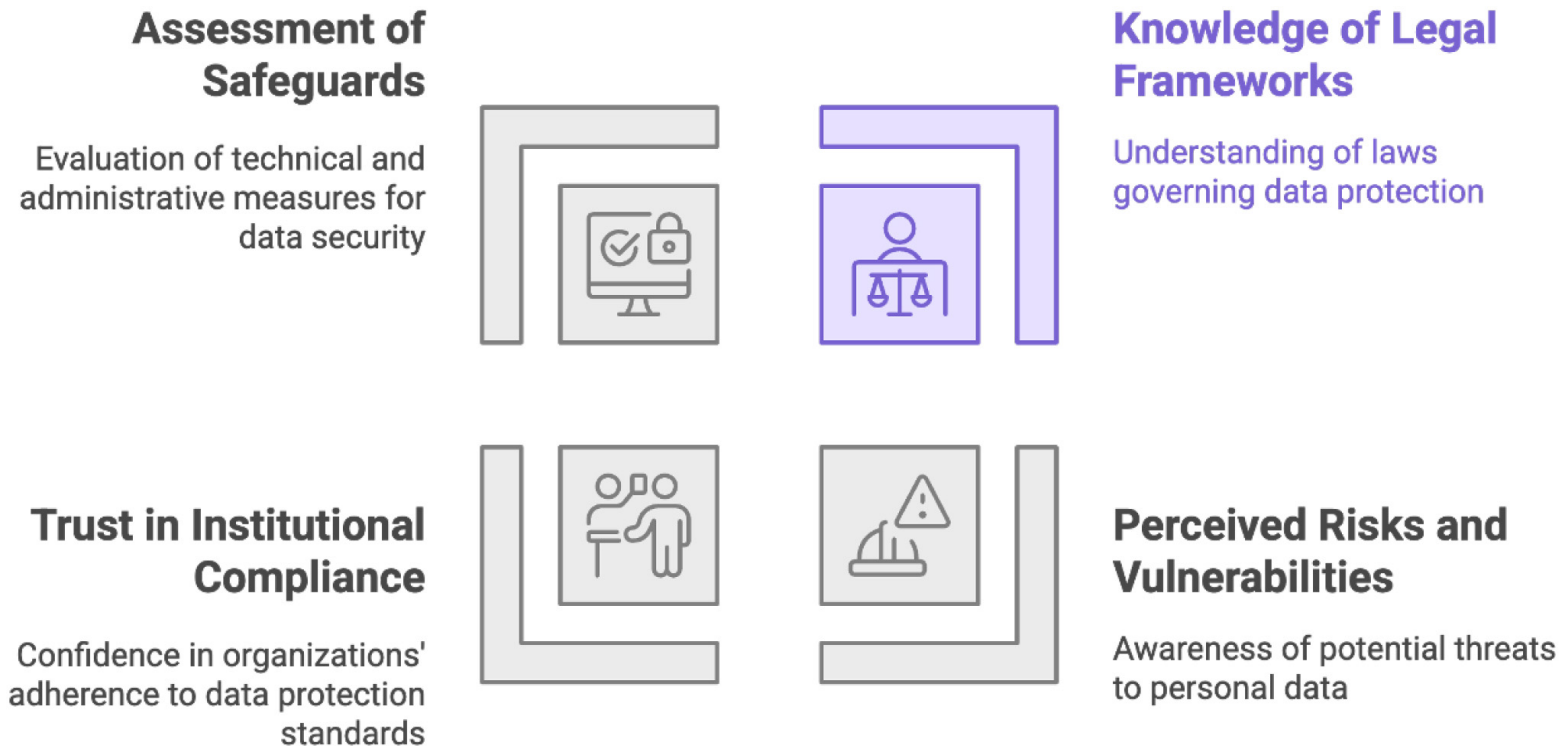
Foundations of data protection perception.

The review of the state of the art revealed a notable gap consisting of limited empirical evidence evaluating the relationship between positive valuation of AI and perceptions regarding personal data protection (Benk et al., 2025) in Mexico City. This absence led to the research question: how are perceptions of artificial intelligence associated with perceptions of personal data protection among university-educated professionals residing in Mexico City in 2025? Consequently, the research analyzed this association by considering four facets that collectively shape citizens' perceptions of data protection: 1) the level of knowledge regarding existing legal frameworks, 2) perceived risks and vulnerabilities, 3) trust in institutional compliance and responsibility, and 4) the assessment of available technical and administrative safeguards (Figure 1).

This study relies on two theories explaining the relationship between AI and data protection constructs: Westin's theory (2003) and Davis's Technology Acceptance Model (TAM, 1989). From Westin's perspective (2003), citizens' perceptions of information control depend on clear regulatory frameworks. Meanwhile, Davis's Technology Acceptance Model (1989) asserts that technological adoption depends on perceived usefulness and ease of use. Recent studies extend this model to the context of AI (Shin, 2021). The convergence of both theories explains why AI, when implemented with robust governance and trust mechanisms, can enhance citizens' perceptions of data protection, reduce uncertainty, and consolidate social acceptance of these technologies.

Based on this theoretical framework, the general hypothesis posited was that AI is positively associated with the perceptions of personal data protection among university-educated professionals residing in Mexico City. Specifically, a more favorable assessment of AI is associated with higher ratings of normative, technical, and institutional instruments designed to preserve privacy.

## 2 Methodology

**Figure 2:**
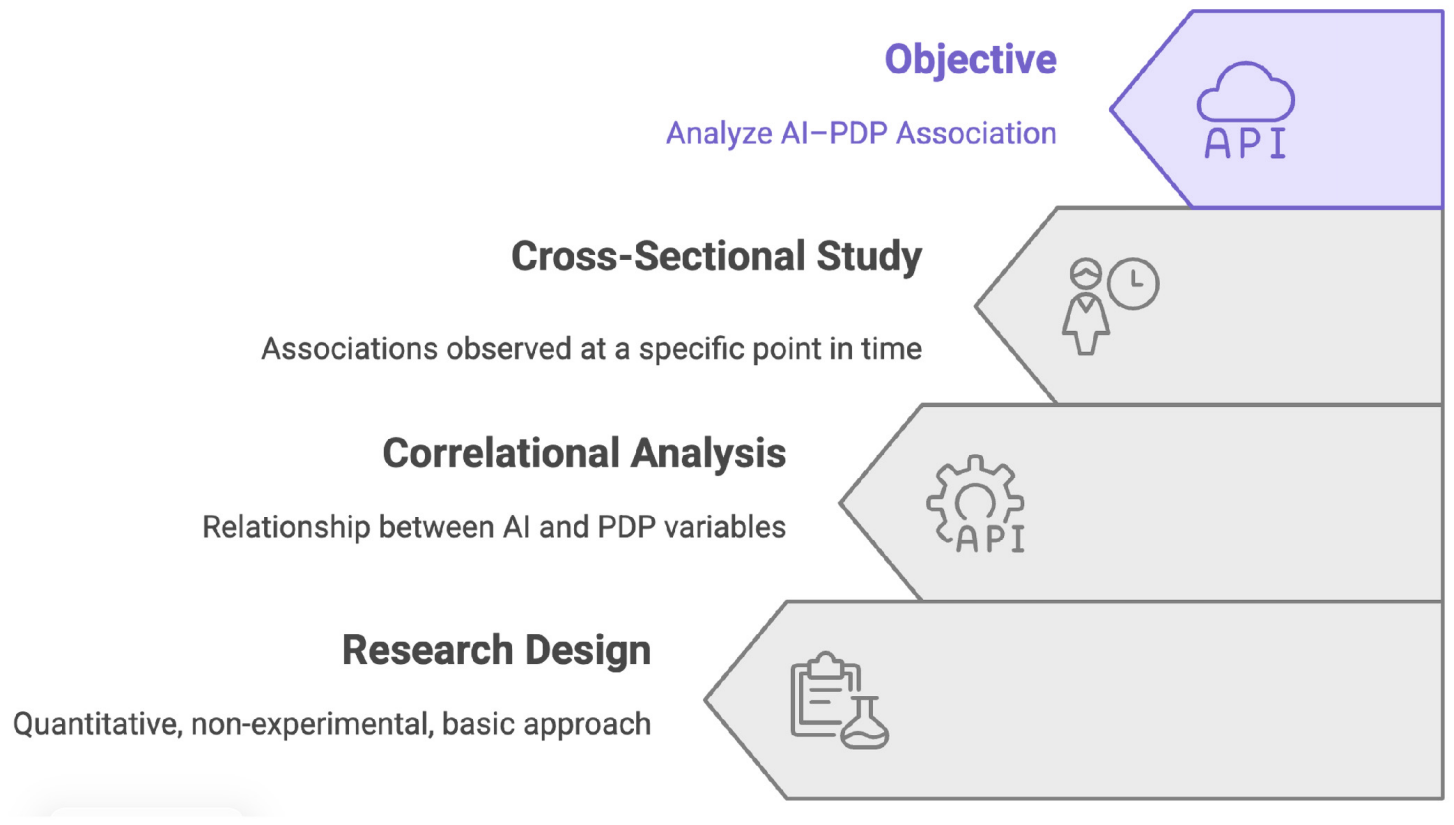
Conceptual framework illustrating the relationship between artificial intelligence and personal data protection, including the analytical dimensions assessed in the study.

The research employed a quantitative, non-experimental, basic approach with a correlational and cross-sectional design, aiming to analyze the relationship between artificial intelligence (AI) and personal data protection (PDP) variables without manipulating them, examining their associations at a specific point in time. The primary objective was to analyze how artificial intelligence is associated with the perception of PDP among university-educated professionals residing in Mexico City in 2025 (Figure 2).

**Figure 3:**
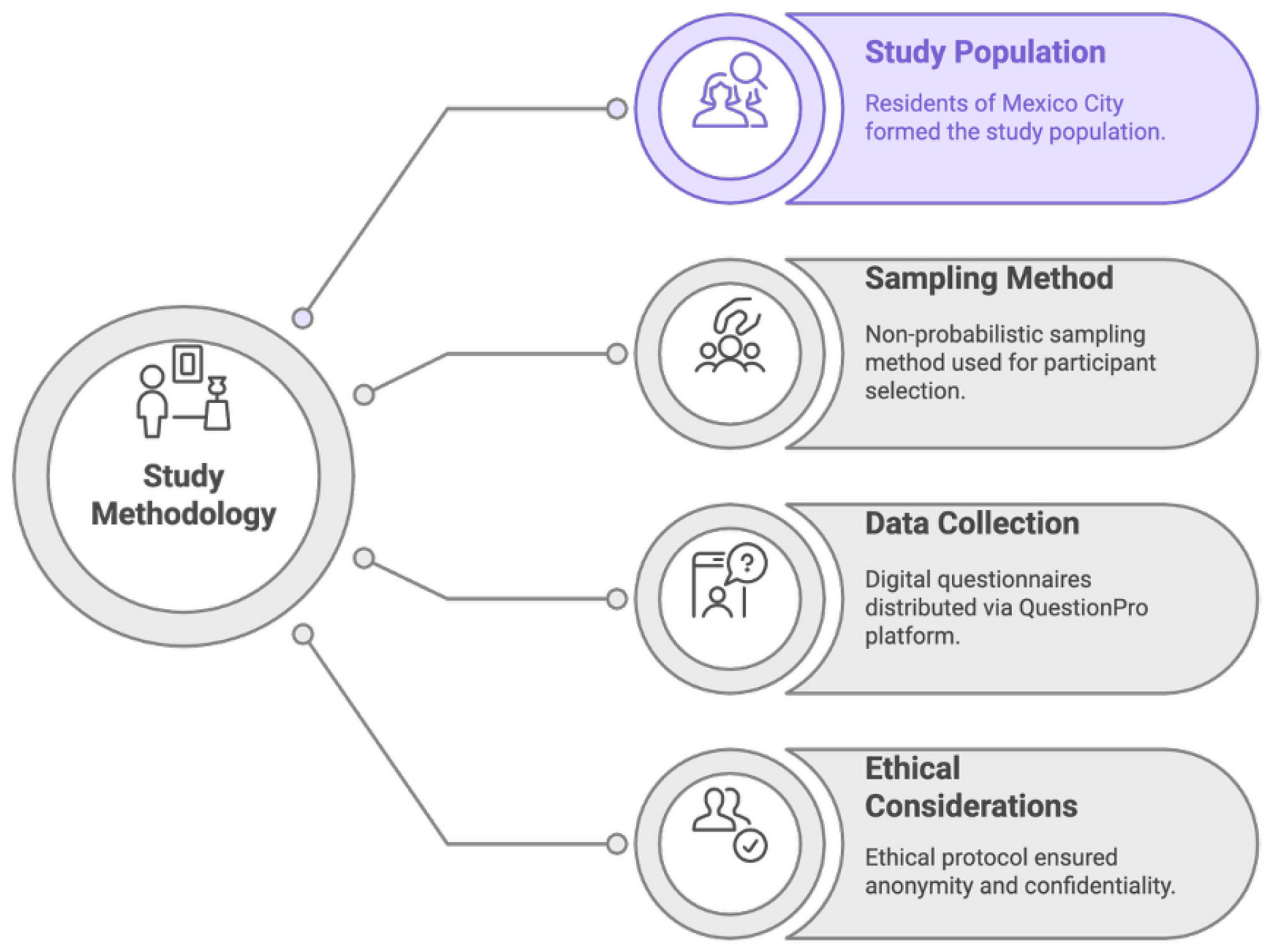
Methodological design of the study, illustrating the quantitative, non-experimental, cross-sectional approach and the main stages of data collection and analysis.

The study population consisted of university-educated professionals residing in Mexico City. From this group, a non-probabilistic sample of 101 participants was drawn. To document the adequacy of the sample size, an a priori statistical power analysis was conducted to estimate the minimum required sample size for correlational testing using Fisher's z-transformation. The analysis assumed a medium expected effect size (*r* = 0.30), a significance level of α = 0.05, statistical power of 0.80, and a two-tailed test. Under these assumptions, the minimum required sample size was estimated at 85 participants. The final sample of 101 respondents therefore exceeded this minimum threshold, ensuring sufficient statistical power for the analyses conducted. Participants completed a structured questionnaire distributed digitally through the QuestionPro platform. Participation was restricted to individuals who were physically residing in Mexico City and who had completed their university education in Mexico City. Only participants who met both criteria during an initial verification stage were subsequently invited to complete the online questionnaire. These inclusion criteria were verified prior to granting access to the survey link. Data collection continued until the minimum sample size required by the a priori power analysis was exceeded, resulting in a final sample of 101 respondents. All participants provided informed consent prior to participation, and the survey was conducted anonymously (Figure 3).

Regarding the recruitment procedure and the composition of the sample, participants were recruited through the online distribution of the survey link, following a convenience-based recruitment approach that enabled voluntary participation. The resulting sample consisted exclusively of professionals with university-level education from diverse fields. This profile was intentionally selected, as professionals with formal higher education are particularly well-positioned to interpret regulatory frameworks, engage with digital technologies, and make informed decisions regarding the use of artificial intelligence and the protection of personal data. This profile was intentionally selected, as higher education has been associated with stronger digital competence and information literacy, which support more informed engagement with digital systems and regulatory and institutional information (Ng, 2012; Spante et al., 2018). In line with ethical considerations related to anonymity and data protection guiding the study and to ensure anonymity and data protection, demographic variables such as age and gender were not collected.

Regarding the data collection instrument (Supplementary Files 1-English Survey, 2-Spanish Survey), it consisted of a structured questionnaire with 24 items, based on a five-point Likert scale (1 = Strongly disagree, 5 = Strongly agree), constructed from a consistency matrix defining study variables, dimensions, and indicators (Supplementary File 3-Data Base). All questionnaire items were formulated in a positive direction; therefore, no reverse-coded items were required. For analytical purposes, composite scores for artificial intelligence and personal data protection were calculated by summing the corresponding item responses rather than computing mean scores. These summed scores were used consistently in all descriptive and inferential analyses conducted in SPSS. The included variables were artificial intelligence and personal data protection, each subdivided into dimensions and indicators.

The 24-item instrument was evenly distributed across the two main constructs analyzed in the study. Artificial intelligence (AI) was measured using 12 items grouped into four dimensions: (i) understanding and knowledge of AI, (ii) use and experience with AI systems, (iii) trust and attitudes toward AI, and (iv) ethical evaluation and perceived responsibility of AI systems, with three items assigned to each dimension. Personal data protection (PDP) was also measured using 12 items organized into four dimensions: (i) knowledge of data protection regulations, (ii) perception of risks and vulnerabilities (for this dimension, higher scores indicate lower perceived risk and vulnerability), (iii) institutional compliance and responsibility, and (iv) Perception of Safeguard, likewise comprising three items per dimension. This structure ensured a balanced representation of each construct and its analytical dimensions.

To clarify the operationalization of the theoretical constructs, representative items from the questionnaire are presented as examples. The construct of artificial intelligence was measured through items assessing perceived understanding, ethical evaluation, and trust in AI systems. For example, respondents were asked to indicate their level of agreement with statements such as: *I understand how artificial intelligence systems use personal data* and *Artificial intelligence systems operate in a transparent and ethical manner*.

Personal data protection was operationalized through items capturing perceptions of rights awareness, including awareness of core data-subject rights recognized in Mexico (ARCO: access, rectification, cancellation, and opposition), risk exposure, institutional responsibility, and safeguards. Illustrative items included statements such as: *I am aware of my rights regarding the protection of my personal data* and *Institutions that use artificial intelligence adequately protect my personal information*. These items allowed the theoretical constructs to be empirically examined through citizens' perceptions, ensuring coherence between conceptual definitions and measurement.

To ensure content validity, the instrument underwent expert judgment validation involving five PhD specialists in AI, data ethics, and research methodology. Instrument reliability was assessed using Cronbach's α coefficient, yielding a value of 0.911, indicating high internal consistency suitable for scientific research purposes (see Table 4). The reported Cronbach's α corresponds to the full 24-item instrument and was used as a global reliability indicator. Internal consistency was evaluated at the level of the full instrument rather than separately for each construct or dimension. Cronbach's α coefficients were not calculated independently for the artificial intelligence (AI) and personal data protection (PDP) constructs, as the primary purpose of the reliability analysis was to verify the overall coherence of the perception-based instrument as a whole. This decision is aligned with the study's correlational and non-experimental design, in which the questionnaire is treated as an integrated measure of related perceptions and the analytical focus is placed on examining associations between aggregated constructs rather than on validating or comparing multidimensional subscales. This approach is consistent with prior peer-reviewed studies employing aggregated Likert-type instruments in correlational and non-experimental research designs, particularly when constructs are analyzed as perception-based measures and the primary objective is to examine associations rather than to validate multidimensional measurement models (Medrano-Sánchez and Ochoa-Tataje, 2024; Medrano-Sánchez et al., 2025). Accordingly, the questionnaire was treated as an integrated, perception-based measurement instrument for correlational analysis, in which items and dimensions were conceptually defined a priori and analytically aggregated to examine relationships between the main study constructs.

**Table 4:** Reliability statistics (pilot test and full sample).

Sample	Cronbach's α	Cronbach's α (standardized items)	Number of items
Pilot test (*n* = 15)	0.911	0.908	24
Full sample (*n* = 101)	0.926	–	24
AI scale (*n* = 101)	0.832	–	12
PDP scale (*n* = 101)	0.921	–	12

In addition to the global reliability estimate, internal consistency was also examined at the construct level using the full study sample (*n* = 101). The Artificial Intelligence (AI) scale, composed of 12 items, demonstrated excellent reliability (Cronbach's α = 0.832), while the personal data protection (PDP) scale, also composed of 12 items, showed good internal consistency (Cronbach's α = 0.921). The full 24-item instrument yielded a Cronbach's α of 0.926, indicating excellent overall reliability (see Table 4). These results confirm the stability and coherence of the measurement instrument used in the correlational analyses.

For descriptive and cross-tabulation purposes, the five-point Likert-scale responses were recoded into three ordinal categories. Specifically, responses of 1 (Strongly disagree) and 2 (Disagree) were classified as “Poor,” response 3 (Neither agree nor disagree) was categorized as “Average,” and responses of 4 (Agree) and 5 (Strongly agree) were classified as “Good.” These categories were used consistently in the cross-tabulation analyses to facilitate the interpretation of perceptions of artificial intelligence and personal data protection.

Once data were collected, statistical analysis was performed using *SPSS-Version 29.0.2.0* software. Initially, data normality was tested with the Kolmogorov–Smirnov test due to the sample size exceeding 50. Subsequently, descriptive and inferential analyses were conducted using Spearman's Rho and Kendall's Tau_b_ correlation coefficients to evaluate the associations between the primary variables and test hypotheses.

The gathered evidence was coded and securely stored, ensuring strictly academic handling in accordance with institutional ethics standards. Data will remain in the custody of responsible researchers for two years to guarantee process integrity and confidentiality.

## 3 Results

This section presents findings from the quantitative analysis applied to a sample of university-educated professionals residing in Mexico City, exploring the relationship between artificial intelligence and personal data protection. Initially, descriptive results obtained through cross-tabulations are presented, enabling preliminary observation of association patterns between the studied variables. Subsequently, specific hypotheses were tested using non-parametric statistics to confirm significant relationships between AI and analyzed dimensions: Legal and Regulatory Knowledge, Perceived Risks and Vulnerabilities, Compliance and Responsibility and, Perception of Safeguard. The obtained results provide consistent empirical evidence discussed in the following section.

### 3.1 Descriptive results

To preliminarily examine the relationship established in the general objective, “analyze how artificial intelligence is associated with personal data protection among university-educated professionals residing in Mexico City in 2025,” Table 5 was constructed through SPSS cross-tabulation. It presents the joint distribution between citizens' perceptions of AI, categorized as poor, average, or good, and their evaluation regarding personal data protection, similarly classified. The intersection of these variables identifies patterns indicating potential associations to be further examined using Spearman's Rho and Kendall's Tau correlation coefficients.

**Table 5:** Cross-tabulation: artificial intelligence and personal data protection.

			Personal data protection			
Independent variable	Level	Measure	Poor	Average	Good	Total
Artificial intelligence	Poor	Count	25	10	0	35
		% of total	24.8%	9.9%	0.0%	34.7%
	Average	Count	8	24	11	43
		% of total	7.9%	23.8%	10.9%	42.6%
	Good	Count	2	6	15	23
		% of total	2.0%	5.9%	14.9%	22.8%
Total		Count	35	40	26	101
		% of total	34.7%	39.6%	25.7%	100.0%

The cross-tabulation presented in Table 5 shows a clear correspondence between respondents' evaluations of artificial intelligence and their perceptions of personal data protection. Negative assessments of AI were predominantly associated with poor evaluations of data protection, whereas positive assessments of AI were more frequently linked to favorable perceptions of data protection. Respondents who rated AI as fair tended to cluster around intermediate evaluations of personal data protection, indicating a neutral or moderate stance. Overall, the distribution reveals a consistent alignment between attitudes toward artificial intelligence and perceptions of personal data protection across categories.

The cross-tabulation presented in Table 6 shows a clear association between respondents' evaluations of artificial intelligence and their self-reported knowledge of laws and regulations related to personal data protection. Lower evaluations of AI were predominantly associated with lower levels of legal and regulatory knowledge, whereas more favorable evaluations of AI corresponded to higher levels of reported legal knowledge. Respondents who assessed AI as fair tended to display intermediate levels of regulatory knowledge. Overall, the distribution indicates a consistent progression whereby knowledge of personal data protection laws increases alongside more positive perceptions of artificial intelligence.

**Table 6:** Cross-tabulation: artificial intelligence and knowledge of law and regulations.

			Knowledge of law and regulations			
Independent variable	Level	Measure	Poor	Average	Good	Total
Artificial intelligence	Poor	Count	19	14	2	35
		% of total	18.8%	13.9%	2.0%	34.7%
	Average	Count	14	14	15	43
		% of total	13.9%	13.9%	14.9%	42.6%
	Good	Count	4	8	11	23
		% of total	4.0%	7.9%	10.9%	22.8%
Total		Count	37	36	28	101
		% of total	36.6%	35.6%	27.7%	100.0%

The cross-tabulation presented in Table 7 shows a clear association between respondents' evaluations of artificial intelligence and their perceived levels of risks and vulnerabilities regarding personal data protection. Lower evaluations of AI were predominantly associated with higher perceived risks and vulnerabilities, whereas more favorable evaluations of AI corresponded to lower perceived levels of risk. Respondents who assessed AI as average tended to report intermediate perceptions of risk. Overall, the distribution indicates a consistent pattern in which perceptions of risk decrease as evaluations of artificial intelligence become more positive.

**Table 7:** Cross-tabulation between artificial intelligence and perceived risks and vulnerabilities.

			Perceived risks and vulnerabilities			
Independent variable	Level	Measure	Poor	Average	Good	Total
Artificial intelligence	Poor	Count	22	13	0	35
		% of total	21.8%	12.9%	0.0%	34.7%
	Average	Count	14	25	4	43
		% of total	13.9%	24.8%	4.0%	42.6%
	Good	Count	1	12	10	23
		% of total	1.0%	11.9%	9.9%	22.8%
Total		Count	37	50	14	101
		% of total	36.6%	49.5%	13.9%	100.0%

The cross-tabulation shown in Table 8 indicates a consistent association between respondents' evaluations of artificial intelligence and their perceptions of institutional Compliance and Responsibility in personal data protection. Lower evaluations of AI were predominantly associated with perceptions of poor Compliance and Responsibility, whereas more favorable evaluations of AI corresponded to higher perceived levels of institutional responsibility. Participants who rated AI as average tended to report intermediate redCompliance and Responsibility. Overall, the distribution reveals a clear pattern in which confidence in institutional compliance increases as evaluations of artificial intelligence become more positive.

**Table 8:** Cross-tabulation between artificial intelligence and compliance and responsibility.

			Compliance and responsibility			
Independent variable	Level	Measure	Poor	Average	Good	Total
Artificial intelligence	Poor	Count	29	6	0	35
		% of total	28.7%	5.9%	0.0%	34.7%
	Average	Count	15	20	8	43
		% of total	14.9%	19.8%	7.9%	42.6%
	Good	Count	2	6	15	23
		% of total	2.0%	5.9%	14.9%	22.8%
Total		Count	46	32	23	101
		% of total	45.5%	31.7%	22.8%	100.0%

The cross-tabulation presented in Table 9 reveals a consistent association between respondents' evaluations of artificial intelligence and their Perception of Safeguard for personal data protection. Lower evaluations of AI were predominantly associated with perceptions of poor safeguards, whereas more favorable evaluations of AI corresponded to higher perceived levels of protection. Participants with average evaluations of AI tended to report intermediate assessments of the Perception of Safeguard dimension. Overall, the distribution indicates that Perception of Safeguard improves progressively as evaluations of artificial intelligence become more positive.

**Table 9:** Cross-tabulation between artificial intelligence and perception of safeguard.

			Perception of safeguard			
Independent variable	Level	Measure	Poor	Average	Good	Total
Artificial intelligence	Poor	Count	30	5	0	35
		% of total	29.7%	5.0%	0.0%	34.7%
		Average	Count	13	24	6	43
	% of total		12.9%	23.8%	5.9%	42.6%
	Good	Count	1	12	10	23
		% of total	1.0%	11.9%	9.9%	22.8%
Total		Count	44	41	16	101
		% of total	43.6%	40.6%	15.8%	100.0%

While these descriptive findings offer a clear basis for identifying patterns, it is necessary to apply inferential statistical tests, such as the Spearman's rho and Kendall's tau coefficients, to confirm whether these associations are statistically significant and to what extent the relationships show consistent strength within the analyzed dataset.

### 3.2 Inferential results

To verify the distribution of the variables included in the study, two normality tests were applied: Kolmogorov–Smirnov (KS) and Shapiro–Wilk (SW), the latter used as a complementary measure despite the sample size exceeding 50 cases (*n* = 101). The null hypothesis (*H*_0_) assumed that the data followed a normal distribution, while the alternative hypothesis (*H*_1_) proposed that the data did not. According to the adopted decision criterion, if the significance value (*p*) is less than 0.05, the null hypothesis is rejected, and it is concluded that the data do not follow a normal distribution, as shown in Table 10.

**Table 10:** Normality tests.

Variable	Kolmogorov–Smirnov^*a*^			Shapiro–Wilk		
	Statistic	df	Sig.	Statistic	df	Sig.
Artificial intelligence	0.226	101	< 0.001	0.803	101	< 0.001
Personal data protection	0.226	101	< 0.001	0.803	101	< 0.001
Legal and regulatory knowledge	0.239	101	< 0.001	0.796	101	< 0.001
Perceived risks and vulnerabilities	0.265	101	< 0.001	0.787	101	< 0.001
Compliance and responsibility	0.289	101	< 0.001	0.772	101	< 0.001
Perception of safeguard	0.277	101	< 0.001	0.781	101	< 0.001

^*a*^ Lilliefors Significance Correction.

Tests were performed on composite variables derived from aggregated Likert-type items. Identical normality statistics for the Artificial Intelligence and Personal Data Protection composite variables reflect highly similar empirical distributions derived from aggregated Likert-type items, rather than duplication or reporting errors.

The results shown in Table 10 indicate that all the variables analyzed yielded significance values below 0.001 in both the Kolmogorov-Smirnov and Shapiro-Wilk tests. This outcome is consistent across the variables: artificial intelligence (*p* < 0.001), personal data protection (*p* < 0.001), as well as the dimensions of legal and regulatory knowledge (*p* < 0.001), perceived risks and vulnerabilities (*p* < 0.001), compliance and responsibility (*p* < 0.001), and perception of safeguards (*p* < 0.001). In all cases, the null hypothesis is rejected, and the alternative hypothesis is accepted, confirming that the data do not follow a normal distribution. This result validates the methodological decision to use non-parametric statistics for inferential analysis, specifically Spearman's rho and Kendall's tau-b correlation coefficients, which do not assume normality and are appropriate for assessing monotonic associations between variables measured on an ordinal scale or when distributional assumptions for parametric correlation are not met.

Although the study hypotheses anticipated positive associations between artificial intelligence and personal data protection and its dimensions, all inferential analyses were conducted and reported using two-tailed significance tests. This decision follows common methodological and reporting standards in correlational research and facilitates transparency and comparability of results. Under the two-tailed criterion, all reported associations remained statistically significant (*p* < 0.001), indicating that the study findings are robust and not dependent on tail specification.

To test the general hypothesis stating that “Artificial intelligence is associated with the protection of personal data among university-educated professionals residing in Mexico City in 2025” the non-parametric tests of Kendall's tau-b and Spearman's rho were applied. These were selected based on the non-normal distribution of the data. Table 11 presents the results: a Kendall's tau-b coefficient of 0.587 and a Spearman's rho coefficient of 0.635, both with a two-tailed significance level of *p* < 0.001, indicating that the relationship between the variables is statistically significant at the 1% level.

**Table 11:** Non-parametric correlations between artificial intelligence and personal data protection.

Correlation type	Independent variable	Measure	Artificial intelligence	Personal data protection
Kendall's Tau-b	Artificial intelligence	Correlation coefficient	1.000	0.587^**^
		Sig. (2-tailed)	.	< 0.001
		*N*	101	101
Spearman's rho	Artificial intelligence	Correlation coefficient	1.000	0.635^**^
		Sig. (2-tailed)	.	< 0.001
		*N*	101	101

^**^ Correlation is significant at the 0.01 level (2-tailed).

A significance level below 0.05 allows for the rejection of the null hypothesis, which stated that no relationship exists between the variables, and supports the acceptance of the alternative hypothesis, which affirms that artificial intelligence is associated with the protection of personal data. In interpretative terms, the Spearman's rho value of 0.635 represents a moderate-to-high positive correlation, while Kendall's tau-b value of 0.587 indicates a moderate positive association. Both results fall within a range that confirms a clear, consistent, and directly proportional relationship between the two variables.

Therefore, the correlation shown in Table 11 suggests that perceptions of AI are not limited to technological functionality but extend toward broader judgments about the protection of digital rights. This not only validates the general hypothesis statistically but also structurally, as the variables are interconnected through interrelated perceptions that shape citizens' judgments regarding the ethical use of data in a context of increasing automation.

To test the first specific hypothesis, which states that “Artificial intelligence is associated with the knowledge of law and regulations regarding the protection of personal data among university-educated professionals residing in Mexico City in 2025,” the non-parametric tests of Kendall's tau-b and Spearman's rho were applied, based on the previously confirmed non-normality of the data. The results are presented in Table 12.

**Table 12:** Non-parametric correlation analysis between artificial intelligence and legal and regulatory knowledge.

Correlation type	Independent variable	Measure	Artificial intelligence	Legal and regulatory knowledge
Kendall's Tau-b	Artificial intelligence	Correlation coefficient	1.000	0.338^**^
		Sig. (2-tailed)	.	< 0.001
		*N*	101	101
Spearman's rho	Artificial intelligence	Correlation coefficient	1.000	0.380^**^
		Sig. (2-tailed)	.	< 0.001
		*N*	101	101

^**^ The correlation is significant at the 0.01 level (2-tailed).

Kendall's tau-b coefficient was 0.338, while Spearman's rho reached a value of 0.380, both with a significance level of *p* < .001. These results allow for the rejection of the null hypothesis and support the acceptance of the alternative hypothesis. In other words, there is a statistically moderate relationship between the two variables. Substantively, a weak-to-moderate positive correlation was identified, meaning that higher evaluations of artificial intelligence are associated with higher levels of perceived knowledge about the legal and regulatory framework related to the protection of personal data.

This connection can be explained through the conceptual structure of the variables. The independent variable includes dimensions such as understanding AI, conscious use of its applications, and attitudes toward its ethical implementation. On the other hand, the “knowledge of law and regulations” dimension considers the ability to identify what constitutes personal data, knowledge of ARCO rights (access, rectification, cancellation, and opposition), and familiarity with existing legislation. The results suggest that individuals with a more informed or positive perception of AI also tend to be better educated or at least more aware of the regulatory framework governing its use. While the relationship is not strong, it is consistent, positioning technological trust as a potential factor associated with legal literacy in digital matters.

These findings support the hypothesis from both a statistical and substantive standpoint: a favorable perception of AI is linked to greater knowledge of the norms that protect personal information. Although causality is not established, a significant pattern is identified, which should be considered in the design of public policies aimed at strengthening citizens' digital and legal education in the face of current technological challenges.

To test the second specific hypothesis, “Artificial intelligence is associated with perceived risks and vulnerabilities regarding the protection of personal data among university-educated professionals residing in Mexico City in 2025,” the non-parametric coefficients of Kendall's tau-b and Spearman's rho were applied, in line with the non-parametric nature of the data identified in the normality analysis. The results, presented in Table 13, show a statistically significant and positive correlation between the two variables.

**Table 13:** Non-parametric correlation analysis between artificial intelligence and perceived risks and vulnerabilities.

Correlation type	Independent variable	Measure	Artificial intelligence	Perceived risks and vulnerabilities
Kendall's Tau-b	Artificial intelligence	Correlation coefficient	1.000	0.500^**^
		Sig. (2-tailed)	.	< 0.001
		*N*	101	101
Spearman's rho	Artificial intelligence	Correlation coefficient	1.000	0.542^**^
		Sig. (2-tailed)	.	< 0.001
		*N*	101	101

^**^ The correlation is significant at the 0.01 level (2-tailed).

The values obtained allow for the rejection of the null hypothesis and, consequently, the acceptance of the alternative hypothesis, confirming the existence of a moderate positive relationship between perceptions of artificial intelligence and perceived risks and vulnerabilities regarding the protection of personal data. The results from both coefficients reflect a moderate positive correlation. Because this dimension was coded so that higher scores indicate lower perceived risk and vulnerability (i.e., a more favorable risk perception), the positive association indicates that as the assessment of artificial intelligence improves, respondents tend to report lower perceived exposure in personal data management. In other words, individuals who evaluate the performance, ethics, and functionality of AI favorably also tend to feel less exposed to threats such as the misuse of information, lack of transparency in data processing, or the absence of effective control mechanisms. In this way, the validation of the specific hypothesis confirms that technologies not only produce operational outcomes but also shape perceptions of safety or risk, which are critical in forming attitudes toward digital governance.

To test the third specific hypothesis, which states that “artificial intelligence is associated with the perception of compliance and responsibility in the protection of personal data among university-educated professionals residing in Mexico City in 2025,” the non-parametric statistics Kendall's tau-b and Spearman's rho were applied, following the methodological criteria established for data that do not follow a normal distribution. The results are presented in Table 14.

**Table 14:** Non-parametric correlation analysis between artificial intelligence and compliance and responsibility.

Correlation type	Independent variable	Measure	Artificial intelligence	Compliance and responsibility
Kendall's Tau-b	Artificial intelligence	Correlation coefficient	1.000	0.600^**^
		Sig. (2-tailed)	.	< 0.001
		*N*	101	101
Spearman's rho	Artificial intelligence	Correlation coefficient	1.000	0.648^**^
		Sig. (2-tailed)	.	< 0.001
		*N*	101	101

^**^ The correlation is significant at the 0.01 level (2-tailed).

According to Table 14, these figures allow us to reject the null hypothesis and, consequently, accept the alternative hypothesis, confirming that there is a statistically significant positive relationship between perceptions of artificial intelligence and the evaluation of compliance and responsibility in the protection of personal data.

The Spearman's rho coefficient indicates a moderately strong positive correlation, while Kendall's tau-b reflects a moderate correlation. This means that as citizens perceive artificial intelligence more positively in terms of understanding, use, ethics, and transparency, they also tend to believe there is greater regulatory compliance and responsibility on the part of the entities managing their personal data. These findings reinforce the notion that technological and institutional perceptions are not independent but are constructed in an integrated manner based on citizens' experiences within the digital ecosystem.

To test the fourth specific hypothesis, which states that “artificial intelligence is associated with the perception of safeguard in the protection of personal data among university-educated professionals residing in Mexico City in 2025,” the non-parametric statistics Kendall's tau-b and Spearman's rho were applied, as defined in the methodological design given the non-normality of the data. Table 15 presents the values obtained for this relationship.

**Table 15:** Non-parametric correlation analysis between artificial intelligence and perception of safeguard.

Correlation type	Independent variable	Measure	Artificial intelligence	Perception of safeguard
Kendall's Tau-b	Artificial intelligence	Correlation coefficient	1.000	0.617^**^
		Sig. (2-tailed)	.	< 0.001
		*N*	101	101
Spearman's rho	Artificial Intelligence	Correlation Coefficient	1.000	0.669^**^
		Sig. (2-tailed)	.	< 0.001
		*N*	101	101

^**^ The correlation is significant at the 0.01 level (2-tailed).

According to Table 15, Kendall's tau-b coefficient was 0.617, while Spearman's rho reached a value of 0.669, both with a two-tailed p-values of *p* < .001. These results support the study hypothesis, indicating a statistically significant and positive relationship between perceptions of artificial intelligence and perception of safeguard in the protection of personal data. In terms of magnitude, both coefficients indicate a moderately strong positive correlation

Taken together, the inferential results obtained through Kendall's tau-b and Spearman's rho confirm the four specific hypotheses proposed in this study. They reveal positive, statistically significant, and consistent relationships between perceptions of AI and each of the dimensions associated with personal data protection.

## 4 Discussion of results

The review of the results showed a clear pattern: the more favorable the assessment of AI, the greater the reported trust in personal data protection mechanisms. The overall association between both variables reached a Spearman coefficient of 0.635, while the specific correlations ranged from 0.338 to 0.669, evidencing positive links of low-moderate to moderate-high intensity depending on the dimension considered. These relationships were consistently observed across the four axes analyzed, confirming both the general and specific hypotheses. In summary, the surveyed population tended to transfer the trust placed in AI systems to the legal and technical structures that protect their personal information, so that technological perception functioned as a relevant factor associated with citizens' judgment regarding security and responsibility in data processing.

The findings of the study showed that a positive assessment of AI was associated with a better perception of personal data protection, in line with privacy theory (Westin, 2003), which holds that control and privacy guarantees are key to generating trust in the processing of personal data. This aligns with previous studies suggesting that a favorable attitude toward AI tends to be related to higher levels of institutional trust and expectations of regulatory compliance and perception of safeguard (Leschanowsky et al., 2024; Orbán and Stefkovics, 2025). However, Benk et al. (2025) argue that there is still a gap in the literature regarding the link between positive attitudes toward AI and perceptions of technical data protection mechanisms.

According to Al-Tkhayneh et al. (2023), AI with weak ethical foundations may lead to discriminatory decisions and risks to user privacy. Similarly, Burton et al. (2024) documented the double-edged nature of AI in terms of privacy, capable of improving data security but also of creating new risks such as massive leaks, algorithmic biases, or intensive surveillance. They emphasized that regulatory frameworks such as the European GDPR or the American CCPA emerged precisely to address these challenges. The correspondence of the results with these perspectives suggests that, as in global studies, public trust in AI was linked to the expectation that strong institutional data protection measures are in place.

In particular, technological trust emerged as a central factor, as noted in the theoretical literature. Benk et al. (2025) stated that the acceptance of AI rests on perceiving systems as predictable, transparent, and legitimate, and the present study provided empirical evidence in that regard; those university-educated professionals residing in Mexico City who viewed AI in a more positive light also assumed that privacy mechanisms were reliable. This observed AI-privacy link coincides with multinational surveys in which attitudes toward AI are shaped by social interpretations of its impact.

The results indicated that a better assessment of AI was associated with greater trust in the institutions responsible for protecting personal data. This relationship can be interpreted in light of the technology acceptance model (TAM) and its extensions, which propose that attitudes toward a technology do not depend exclusively on its practical usefulness, but also on perceptions of clarity, ease of use, and ethical management (Davis, 1989; Shin, 2021). These factors are moderated by the institutional context, especially in emerging technologies such as AI, where trust acts as an enabling mechanism that goes beyond basic perceived usefulness (Venkatesh et al., 2003). Such perceptions are associated with acceptance and with expectations that privacy mechanisms exist, such as clear regulations and audits. At the same time, recent studies reaffirm that trust constitutes a universal axis for the adoption of AI, by linking technological innovation with strong data protection guarantees and ethical principles (Kashiramka et al., 2024; Wirtz et al., 2022). However, this relationship is not homogeneous in contexts where regulatory frameworks are incipient; observed trust is more fragile and discontinuous.

Nevertheless, divergences also emerged when compared with previous studies, attributable to the Latin American sociotechnical context and the levels of digital and institutional literacy. The literature had already pointed out that countries in the region adopted laws inspired by the GDPR, but a normative mosaic persists whose effectiveness depends on interstate coordination (Contreras, 2024; Ramiro, 2022). In accordance with that panorama, individuals showed moderate and conditional trust: the positive correlation between perception of AI and assessment of data protection was significant, but it did not imply that everyone had deep knowledge of their rights or unrestricted trust in institutions. In fact, the correlation coefficient between the assessment of AI and regulatory knowledge was lower than that observed with the general perception of protection, indicating that part of the sample trusted AI without necessarily being fully informed about privacy laws.

This difference suggests an association related to unequal levels of digital literacy and legal knowledge, a factor already highlighted in Latin American literature. Chavez and Vizuete-Sandoval (2025) emphasize that in the region, technical capacities and academic production in AI are concentrated in a few countries, leaving gaps in evidence on public perception of these technologies. In this regard, this study covers part of that empirical gap in Mexico, but the results reflected that asymmetries persist. Some citizens internalized the global discourse of trust in AI, while others showed reservations likely linked to a lack of information or experiences of data protection failures.

Likewise, local sociopolitical conditions help explain divergences from studies in different contexts. Filgueiras (2023) found that the strength of national AI policies in Latin America depends on the type of political regime and governance model in each country. In the case of Mexico, a young democracy with relatively recent personal data frameworks, it is understandable that the citizen trust in AI detected is cautious and subject to seeing tangible compliance outcomes. (Juárez-Merino 2025) had already warned that even pioneering cities like Mexico City face a persistent digital divide and the risk of indiscriminate technological surveillance that can undermine public trust. In line with that observation, the findings of this study suggest that, although respondents showed optimism regarding the potential of AI, recognizing its “transformative capacity” in urban services Juárez-Merino, (2025), such optimism was tempered by the latent concern that institutions truly be held accountable and protect privacy. This relative caution marks a difference from studies in more regulated environments such as Europe, while in Mexico, the detected trust relied more on expectations than on legal certainties. Therefore, it is relevant to redirect data protection law toward automated decision-making at the global level (Demetzou et al., 2023).

The literature suggests measures to close the gap, as noted by Alashqar et al. (2025), who emphasize that in smart cities, constant citizen audits and algorithmic transparency mechanisms are needed to sustain public legitimacy. In line with this, the results obtained indicate that, in order to convert incipient trust into full trust, it will be necessary to strengthen the population's critical digital literacy and the institutional capacity to be accountable. Only then can the observed convergences with global studies, such as the expectation of privacy and security in the era of AI, be translated into local realities, overcoming current divergences arising from differences in sociotechnical context, level of regulation, and citizens' digital culture.

Likewise, the study revealed the positive link between perception of AI and trust in privacy, in contexts of emerging regulation where the detected trust was more cautious and conditional. In other words, it was shown that citizens displayed optimism regarding the potential of AI, moderated by uncertainty about the real effectiveness of institutions to protect their data. This finding aligns with previous studies by authors such as Al-Tkhayneh et al. (2023) and Burton et al. (2024), but in contexts with already consolidated regulatory frameworks, reinforcing the notion that the acceptance of intelligent technologies goes hand in hand with the expectation of strong privacy guarantees.

## 5 Implications

### 5.1 Theoretical implications

The study fills the theoretical gap by identifying a statistically significant association between the evaluation of AI and the perception of data protection, representing a relevant advance in the understanding of these constructs in the context of emerging regulatory frameworks in Mexico.

The research adopts a theoretical framework based on Westin's privacy theory and the Technology Acceptance Model (TAM) and its extensions. This framework not only allows for the analysis of the relationship between institutional trust, data protection, and technological acceptance, but also shows how they interact in contexts where the perception of governance is decisive. The theoretical implications are profound, since they broaden the understanding of the phenomenon beyond the traditional constructs of TAM because they incorporate privacy and institutional trust, dimensions rarely addressed in the literature. This provides a robust basis for future research in the social and technological field, with the potential to explore new explanatory models of emerging technology adoption under different frameworks.

The research interweaves for the first time trust in data protection institutions with the acceptance of AI. The findings show that trust in organizations is associated with lower levels of perceived uncertainty and higher willingness to use AI. This suggests that, beyond usefulness and ease of use, classical pillars of TAM, technological acceptance is associated with the institutional framework perceived as guaranteeing security in data processing.

### 5.2 Practical implications

First, the findings are associated with guidance for public policies focused on AI governance, prioritizing the protection of personal data. The findings allow policymakers to identify regulatory gaps for the establishment of guidelines that strengthen citizen trust in the use of technology.

Second, and in the business context, the results are associated with practical criteria for the implementation of AI systems aligned with ethical principles and responsible functionality. Institutions can make use of the findings to integrate control mechanisms that reduce uncertainty in data management, thus improving innovation and acceptance of their services.

Finally, the study is associated with concrete guidelines for those involved in AI with concrete guidelines to incorporate designs that prioritize aspects such as data security and privacy. This not only optimizes the protection of sensitive information but also reinforces user trust.

## 6 Conclusions

First, the study made it possible to determine that artificial intelligence is directly associated with citizens' perception of personal data protection in Mexico City. It was identified that people who positively value the use of artificial intelligence also tend to have a more favorable perception of the existing mechanisms to safeguard their personal information. This association is reflected not only in legal knowledge but also in the perception of risks, institutional compliance, and the existence of safeguards.

Second, it is concluded that there is a statistically significant association between artificial intelligence and the level of knowledge that the inhabitants of Mexico City have about the laws and regulations related to personal data protection. The empirical evidence suggests that the more interaction with and positive valuation of artificial intelligence, the greater the declared level of knowledge about the legal frameworks that regulate the use and protection of personal information. This relationship highlights that technology is associated with higher levels of awareness regarding legal frameworks and digital rights.

Third, artificial intelligence is significantly associated with the perception of risks and vulnerabilities associated with personal data protection. The results show that as people perceive the use and functioning of artificial intelligence more positively, they tend to consider the risks related to manipulation, leakage, or misuse of their personal information to be lower. This trend shows that the trust in technology is associated with variations in the perception of the risk environment.

Fourth, it is concluded that artificial intelligence is directly associated with citizens' perception of institutional compliance and responsibility in matters of personal data protection. Participants who expressed a positive valuation of artificial intelligence also expressed greater confidence that public and private entities comply with protection standards and act responsibly in handling personal information. This perception reveals that trust in intelligent technologies tends to extend to those who implement and regulate them, being associated with more favorable expectations toward institutional practices. In this context, these findings highlight the relevance of examining the relationship between technological innovation and institutional responsibility within frameworks of data protection and regulatory compliance.

Finally, it was concluded that artificial intelligence is significantly associated with citizens' perception of the safeguarding of personal data. The results show that people who positively value the use of artificial intelligence tend to trust that there are effective technical and regulatory mechanisms to protect their personal information. This perception of protection does not arise from detailed technical knowledge, but from a generalized trust in the digital environment and in the responsible institutions. Therefore, artificial intelligence is associated with higher levels of trust in data protection systems.

## 7 Limitations and future research

First, the study employed a cross-sectional design for data collection, which reflects only a specific moment in time. In this regard, future research could address longitudinal studies, which would allow the analysis of how citizens' perceptions change over time and how the observed associations may vary across periods.

Second, the sample is limited to Mexico City. In this regard, comparisons with other Latin American cities would help determine whether the patterns observed in Mexico City are replicated in different sociocultural contexts.

Third, the study relied on a convenience sample composed exclusively of professionals with university-level education. Given the digital mode of recruitment and the voluntary nature of participation, it cannot be ruled out that individuals with higher familiarity or engagement with digital technologies and artificial intelligence may have been more likely to respond. Although no demographic or background variables such as age, professional sector, or level of digital exposure were collected, this sampling approach may introduce self-selection bias and should be considered when interpreting the results. While this profile was appropriate for exploring informed perceptions regarding artificial intelligence and personal data protection, it may limit the generalizability of the findings to populations with different educational backgrounds or levels of professional expertise. Additionally, because the main constructs were captured through a single self-report instrument at one time point, the observed relationships may be affected by respondent-level tendencies and the shared measurement setting. While this approach is appropriate for exploratory correlational research on perceptions, it warrants a cautious interpretation of the strength of the associations. Therefore, the findings are best understood as evidence of perceived co-variation rather than causal influence. In addition, the questionnaire was designed and analyzed as an integrated, perception-based instrument for correlational purposes rather than as a psychometric scale intended for factorial validation. Accordingly, the reported findings should be interpreted as associations between aggregated perceptions of artificial intelligence and personal data protection, and not as evidence of causal effects or as validation of independent measurement models.

Fourth, the absence of sociodemographic and contextual covariates limits the ability to account for potential confounding effects. Future studies could incorporate relevant control variables (e.g., age, education level, or professional background) to examine these associations using multivariate models (e.g., regression), thereby strengthening the robustness and explanatory scope of the findings.

Additionally, future research could further strengthen this line of inquiry by complementing the present empirical evidence with a focused review of ongoing legislative reform initiatives related to personal data protection and artificial intelligence within Mexico's Legislative Information System. Such a normative perspective would help contextualize how evolving legal debates and regulatory reforms may shape citizens' expectations and trust in data protection in AI-intensive environments.

Finally, the incorporation of qualitative approaches, such as in-depth interviews or focus groups, would serve to capture perceptual nuances and complement the findings of the present study.

## Data Availability

The raw data supporting the conclusions of this article will be made available by the authors, without undue reservation.
