# Supplementary material for: Between innovation and risk: artificial intelligence and data protection in digital Mexico
Source: Front Artif Intell. 2026 May 13;9:1716108. doi: 10.3389/frai.2026.1716108 (PMC13212271; doi:10.3389/frai.2026.1716108)
Supplement: Supplementary file 2 [file Data_Sheet_1.pdf]

Supplementary Material

SUPPLEMENTARY FILE 1- ENGLISH SURVEY

ITEMS		SCALE				
**Independent Variable – Artificial Intelligence**		**SD**	**D**	**U**	**A**	**SA**
**Dimension 1: Knowledge and understanding of AI**						
1.	I have a clear understanding of what Artificial Intelligence is and how it is applied in different areas.					
2.	I am fully aware of the information I provide when I interact with AI systems.					
3.	I understand the advantages and limitations that come with using AI.					
**Dimension 2: Use and Experience with AI**						
4.	I frequently use AI-based applications or services in my daily life.					
5.	I intentionally provide data to AI systems, acknowledging that doing so can improve the experience they offer.					
6.	I find it easy to use technologies that employ AI.					
**Dimension 3: Trust and Attitude toward AI**						
7.	I trust that AI provides results or suggestions with a high degree of accuracy.					
8.	I feel that platforms that use AI clearly inform me about how they collect and process my data.					
9.	I am confident that the AI I use will not use my information inappropriately or with harmful biases.					
**Dimension 4: Ethics and Responsibility in AI**						
10.	I believe that AI developers are fulfilling their obligation to explain to us why and for what purposes they use our data.					
11.	I believe that both the government and companies would take responsibility if any improper use of AI were to affect my personal data.					
12.	I support the creation of laws and specialized regulatory bodies to oversee the proper use of Artificial Intelligence.					

ITEMS		SCALE				
**Independent Variable – Artificial Intelligence**		**SD**	**D**	**U**	**A**	**SA**
**Dimension 1: Knowledge and understanding of AI**						
1.	I have a clear understanding of what Artificial Intelligence is and how it is applied in different areas.					
2.	I am fully aware of the information I provide when I interact with AI systems.					
3.	I understand the advantages and limitations that come with using AI.					
**Dimension 2: Use and Experience with AI**						
4.	I frequently use AI-based applications or services in my daily life.					
5.	I intentionally provide data to AI systems, acknowledging that doing so can improve the experience they offer.					
6.	I find it easy to use technologies that employ AI.					
**Dimension 3: Trust and Attitude toward AI**						
7.	I trust that AI provides results or suggestions with a high degree of accuracy.					
8.	I feel that platforms that use AI clearly inform me about how they collect and process my data.					
9.	I am confident that the AI I use will not use my information inappropriately or with harmful biases.					
**Dimension 4: Ethics and Responsibility in AI**						
10.	I believe that AI developers are fulfilling their obligation to explain to us why and for what purposes they use our data.					
11.	I believe that both the government and companies would take responsibility if any improper use of AI were to affect my personal data.					
12.	I support the creation of laws and specialized regulatory bodies to oversee the proper use of Artificial Intelligence.					
