# Supplementary material for: Between innovation and risk: artificial intelligence and data protection in digital Mexico
Source: Front Artif Intell. 2026 May 13;9:1716108. doi: 10.3389/frai.2026.1716108 (PMC13212271; doi:10.3389/frai.2026.1716108)
Supplement: Supplementary file 3 [file Data_Sheet_2.pdf]

Supplementary Material

SUPPLEMENTARY FILE 1- SPANISH SURVEY

Table S1. Encuesta sobre la **Inteligencia artificial** y la protección de datos

ITEMS	Variable Independiente – Inteligencia Artificial	MD	ED	I	DA	MA
**Dimensión 1: Conocimiento y comprensión de la IA**						
1	Tengo un entendimiento claro de lo que es la Inteligencia Artificial y cómo se aplica en diferentes ámbitos.					
2	Soy plenamente consciente de la información que proporciono cuando interactúo con sistemas de Inteligencia Artificial.					
3	Comprendo las ventajas y las limitaciones que puede tener la Inteligencia Artificial al momento de utilizarla.					
**Dimensión 2: Uso y experiencia con la IA**						
4	Hago uso frecuente de aplicaciones o servicios basados en Inteligencia Artificial en mi vida diaria.					
5	Entrego datos de forma deliberada a los sistemas de IA, reconociendo que esto puede mejorar la experiencia que ofrecen.					
6	Me resulta sencillo manejar las tecnologías que emplean Inteligencia Artificial cuando las utilizo.					
**Dimensión 3: Confianza y actitud hacia la IA**						
7	Confío en que la Inteligencia Artificial brinda resultados o sugerencias con un alto nivel de exactitud.					
8	Percibo que las plataformas que usan IA me informan de manera clara cómo recopilan y procesan mis datos.					
9	Me siento seguro de que la IA que empleo no utilizará mi información de forma inadecuada ni con sesgos perjudiciales.					
**Dimensión 4: Ética y responsabilidad en la IA**						
10	Considero que los desarrolladores de IA están cumpliendo con su obligación de explicarnos por qué y para qué utilizan nuestros datos.					
11	Creo que tanto el Estado como las empresas asumen su responsabilidad si se diera un uso indebido de la IA que afecte mis datos personales.					
12	Apoyo la creación de leyes y entidades regulatorias especializadas para supervisar el adecuado uso de la Inteligencia Artificial.					

Table S2. Encuesta sobre la Inteligencia artificial y la **protección de datos**

ITEMS	Variable dependiente – Protección de datos personales	MD	ED	I	DA	MA
**Dimensión 1: Conocimiento de derecho y normativa**						
1	Reconozco la existencia de normas específicas que protegen mis datos personales en el entorno digital.					
2	Sé distinguir qué tipo de información se considera un dato personal y por qué requiere protección.					
3	Estoy al tanto de mis derechos para acceder, rectificar, cancelar u oponerme al tratamiento de mis datos personales.					
**Dimensión 2: Riesgos y vulnerabilidades percibidas**						
4	Estoy confiado en que la IA no cruzará ni inferirá mis datos sin contar previamente con mi autorización.					
5	Siento que mi información personal está adecuadamente resguardada y no me considero vulnerable ante prácticas indebidas de IA.					
6	Estoy tranquilo respecto a que la IA no elaborará perfiles de mi conducta que puedan afectar mi privacidad.					
**Dimensión 3: Cumplimiento y responsabilidad**						
7	Tengo la certeza de que las instituciones gubernamentales supervisan de forma eficaz a quienes usan IA para proteger mis datos.					
8	Considero que las empresas que recaban datos personales son realmente responsables y cumplen con la normativa de protección de datos.					
9	Estoy dispuesto a presentar una queja o denuncia si considero que han usado mis datos sin respetar mi privacidad.					
**Dimensión 4: Percepción de salvaguarda**						
10	Conozco algunas herramientas o procedimientos como el cifrado y la eliminación segura de datos que ayudan a proteger mi información.					
11	Confío en que los proveedores de IA cumplen con las regulaciones vigentes y poseen la capacidad para prevenir hackeos o brechas de seguridad.					
12	Pienso que los sistemas de IA cuentan con mecanismos tecnológicos suficientes para evitar hackeos o brechas de seguridad.					
